# Development and Characterization of Microsatellite Markers for the Cape Gooseberry *Physalis peruviana*

**DOI:** 10.1371/journal.pone.0026719

**Published:** 2011-10-21

**Authors:** Jaime Simbaqueba, Pilar Sánchez, Erika Sanchez, Victor Manuel Núñez Zarantes, Maria Isabel Chacon, Luz Stella Barrero, Leonardo Mariño-Ramírez

**Affiliations:** 1 Plant Molecular Genetics Laboratory, Center of Biotechnology and Bioindustry (CBB), Colombian Corporation for Agricultural Research (CORPOICA), Bogota, Colombia; 2 Facultad de Agronomía, Universidad Nacional de Colombia, Bogotá, Colombia; 3 PanAmerican Bioinformatics Institute, Santa Marta, Magdalena, Colombia; 4 Computational Biology Branch, National Center for Biotechnology Information, National Library of Medicine, National Institutes of Health, Bethesda, Maryland, United States of America; Georgia Institute of Technology, United States of America

## Abstract

*Physalis peruviana,* commonly known as Cape gooseberry, is an Andean *Solanaceae* fruit with high nutritional value and interesting medicinal properties. In the present study we report the development and characterization of microsatellite loci from a *P. peruviana* commercial Colombian genotype. We identified 932 imperfect and 201 perfect Simple Sequence Repeats (SSR) loci in untranslated regions (UTRs) and 304 imperfect and 83 perfect SSR loci in coding regions from the assembled *Physalis peruviana* leaf transcriptome. The UTR SSR loci were used for the development of 162 primers for amplification. The efficiency of these primers was tested via PCR in a panel of seven *P. peruviana* accessions including Colombia, Kenya and Ecuador ecotypes and one closely related species *Physalis floridana*. We obtained an amplification rate of 83% and a polymorphic rate of 22%. Here we report the first *P. peruviana* specific microsatellite set, a valuable tool for a wide variety of applications, including functional diversity, conservation and improvement of the species.

## Introduction

*Physalis peruviana* commonly known as Cape gooseberry or golden berry is an Andean tropical fruit from the *Solanaceae* family native to South American countries including Colombia, Ecuador and Peru. *Physalis peruviana* grows wild in various parts of the Andes, typically 2,200 meters above sea level. The Cape gooseberry was known to the Incas but their origins are not clear, after Christopher Columbus the Cape gooseberry was introduced into Africa and India [1]. In Colombia, over the last three decades, *P. peruviana* went from being a neglected species to be the most promissory and successful exotic fruit for national and international markets; thus, since 1991, the Cape gooseberry market has been growing annually and in 2007 exports brought USD 34 million into the country. The main consumers of the Colombian Cape gooseberry are Europe with 97%, along with Asia and the United States with the remaining 3% [2]. The commercial interest in this fruit has grown due to its nutritional properties related to high vitamins content, minerals and antioxidants as well as its anti-inflammatory, anti-cancer and other medicinal properties [3], [4], [5], [6], [7], [8].

Despite growing interest in the Cape gooseberry, little is known about its genetic diversity and population structure. The collections kept in germplasm banks have been partially evaluated for morphologic and agronomic traits [9], [10], [11]. Although it has been reported that Cape gooseberry is a diploid species with 2n = 48 [12]; different chromosome numbers might exist among genotypes since 2n = 24 has been reported for wild ecotypes, 2n = 32 for the cultivated Colombia ecotype and 2n = 48 for the cultivated Kenya ecotype [13]. The genetic diversity of the Cape gooseberry at the molecular level has been poorly studied, to our knowledge there is only one report applying dominant markers RAMs (Random Amplified Microsatellites) in 43 individuals from five geographical regions in Colombia suggesting high heterozigocity and genetic diversity [14]. Additionally, in our experience, the use of heterologous microsatellite markers previously developed for several other *Solanaceae* species have not been successful in identifying polymorphic markers in Cape gooseberry.

Microsatellites or SSRs are defined as highly variable DNA sequences composed of tandem repeats of 1–6 nucleotides with co-dominant inheritance which have become the markers of choice for a variety of applications including characterization and certification of plant materials, identification of varieties with agronomic potential, genetic mapping, assistance in plant-breeding programs, among others [15], [16], [17], [18], [19]. However, no SSR markers specific for *P. peruviana* have been developed. The genetic analysis with microsatellites is simple and robust, although their identification and development present significant challenges in emerging species [16], [20]. According to the origin of the sequences used for the initial identification of simple repeats, SSRs are divided in two categories: Genomic SSRs which are derived from random genomic sequences and EST-SSRs derived from expressed sequence tags or from coding sequences. Genomic SSRs are not expected to have neither genic function nor close linkage to transcriptional regions, while EST-SSRs and coding-SSRs are tightly linked with functional genes that may influence certain important agronomic characters. The *de novo* identification of simple sequence repeats has usually involved large-scale sequencing of genomic, SSR-enriched genomic or EST libraries, which are expensive, laborious and time-consuming. Next generation sequencing technologies have enabled rapid identification of SSR loci derived from ESTs which can be identified in any emergent species [17], [19], [21].

The goal of the present study was to identify polymorphic SSR loci using the assembled leaf transcriptome sequences from a commercial Colombian ecotype of *P. peruviana* developed in our laboratory (http://www.ncbi.nlm.nih.gov/bioproject/67621). Imperfect as well as perfect repeat searches in non-coding or untranslated regions (UTRs) were performed. From these loci, primers were designed for amplification of UTR SSR loci. The effectiveness of these primers was tested via PCR in seven *P. peruviana* accessions, among them, the ecotypes Colombia, Kenya and Ecuador, as well as one closely related species *Physalis floridana*. The molecular markers developed here are valuable tools for assessing functional diversity, aid in species conservation and plant breeding programs.

## Materials and Methods

### SSR loci identification and marker development

A collection of *Physalis peruviana* leaf transcript sequences was used as the source for SSR development (Transcriptome Shotgun Assembly (TSA) Database, GenBank Accession numbers JO124085-JO157957). The transcripts were compared for sequence similarity with the non-redundant protein sequences database from NCBI using BLASTX. SSR loci were searched in both coding and non-coding sequences. Candidate SSR loci were identified using Phobos [22] in both coding and non-coding sequences using perfect and imperfect repeat searches with a minimum length of 18 bp for dinucleotides, 24 bp for tri and tetranucleotides, 30 bp for pentanucleotides and 36 bp for hexanucleotide repeats.

### Primer design and amplification of SSR loci by PCR

Primer3 version 0.4.0 [23] was used to design primers for microsatellite amplification in *P. peruviana*. In addition, the oligocalculator - SIGMA Aldrich (http://www.sigma-genosys.com/calc/DNACalc.asp) was used to predict secondary structures (i.e. hairpins, primer dimers) for each primer pair designed. To determine the success of the microsatellite primer design, we carried out PCR tests to amplify the SSR loci in seven *P. peruviana* accessions (including Kenya, Ecuador and Colombia ecotypes) and one *Physalis floridana* accession, a closely related species (Table 1). The following PCR conditions were used: 1X PCR buffer: 1.5 to 3 mM MgCl_2_ depending on the primer pair, 0.2 µM dNTPs, 0.2 to 0.3 µM of each primer (depending on the primer pair), 0.05 U/µl *Taq* polymerase and 25 ng of genomic DNA, in a 15 µl reaction volume. The temperature conditions were 95°C for 3 minutes followed by 35 cycles of 95°C for 30 seconds, 50 to 52°C (depending on the primer pair) for 30 seconds and 72°C for 90 seconds, and a final extension of 72°C for 8 minutes. The PCR amplification products were analyzed by polyacrylamide gel electrophoresis (PAGE).

**Table 1:** Plant material used for SSR development and characterization.

Species	Work Code	Accession/Common Name	Accession Code	Origin	
				Source/region	Country
*P. peruviana*	1	ILS 3804*	09U086-1	CORPOICA/Ambato	Ecuador
*P. peruviana*	2	Ecotype Kenia	09U215-1	Universidad de Nariño/^+^NA	Colombia
*P. peruviana*	3	Ecotype Colombia	09U216-1	Universidad de Nariño/NA	Colombia
*P. floridana*	4	ILS 1437*	09U139-1	Botanical Garden of Birmingham/NA	U.K.
*P. peruviana*	5	Novacampo (commercial)	09U 274-1	CORPOICA/Cundinamarca	Colombia
*P. peruviana*	6	ILS 3807*	09U089-1	CORPOICA/Antioquia	Colombia
*P. peruviana*	7	ILS 3826*	09U108-1	CORPOICA/Antioquia	Colombia
*P. peruviana*	8	ILS 3817*	09U099-1	CORPOICA/Caldas	Colombia

ILS* = Introduction maintained at La Selva Research Center, CORPOICA; NA = Not available; ^+^NA = Not available (*in vitro* propagated material).

### Gene Ontology analysis of SSR loci

A gene ontology (GO) analysis was performed using blast2go [24] with the assembled transcript sequences containing the 30 polymorphic SSRs described here. These sequences were compared with the UniProtKB/Swiss-Prot database with a cutoff e-value of 1×10^−5^.

## Results

### Identification of SSR loci in *P. peruviana*

A total of 1,520 SSR loci were identified and a large fraction were located in UTRs (74%) as compared to coding sequences (CDS) with 26%. The highest number of SSR loci found contained trinucleotide and hexanucleotide repeats with 544 (36%) and 530 (35%) respectively (Table 2).

**Table 2:** SSR loci identified in *Physalis peruviana* leaf Expressed Sequence Tags (ESTs).

Repeat Type	Perfect			Imperfect				Frequency
	CDS	UTRs	Total	CDS	UTRs	Total		
Dinucleotide	-	34	34	2	98	100	**134**	8%
Trinucleotide	36	81	117	178	249	427	**544**	36%
Tetranucleotide	1	16	17	13	69	82	**99**	7%
Pentanucleotide	-	6	6	47	160	207	**213**	14%
Hexanucleotide	46	64	110	64	356	420	**530**	35%
**Total**	**83**	**201**	**284**	**304**	**932**	**1236**	**1520**	**-**
**Frequency**	6%	13%	**19%**	20%	61%	**81%**		

The number of SSR loci identified at coding sequences (CDS) and Untranslated Regions (UTRs) by using perfect and imperfect repeat search criteria.

### Microsatellite primer design and PCR analysis

**Figure 1:**
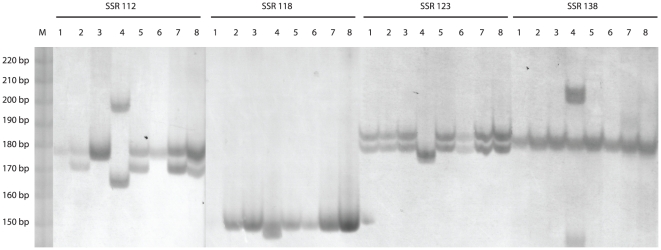
SSR alleles in eight *Physalis* genotypes and four polymorphic loci. The polymorphic SSR loci were visualized in 6% polyacrylamide gels, samples 1–8 correspond to the work code shown in Table 1. M = Molecular size marker, 10 bp DNA Ladder (Invitrogen, Carlsbad, CA).

The SSR loci selected for primer design were located at UTRs and identified with an imperfect repeat search to increase the probabilities for finding polymorphisms within the individuals analyzed. Using this strategy a total of 162 primers pairs were designed. A successful PCR amplification was obtained for 138 (83%) of the 162 primers designed from microsatellite loci using seven *P. peruviana* and one *P*. *floridana* genotype (Table 1). Polymorphisms among the eight genotypes were observed for 30 (22%) loci whereas the remaining 108 loci were monomorphic (Figure 1, Tables 3 and 4).

**Table 3:** Polymorphisms in *Physalis peruviana* SSR loci.

SSR Type	Polymorphic	Monomorphic	Total
Dinucleotide	19	53	72
Trinucleotide	10	39	49
Tetranucleotide	-	5	5
Pentanucleotide	1	1	2
Hexanucleotide	-	10	10
Total	30	108	138

**Table 4:** Allelic variation in 30 *Physalis peruviana* SSR loci.

Polymorphic loci	Forward primer (5′-3′)	Reverse primer (5′-3′)	PCR conditions			Alleles (pb)			Repeat type	Location
			Primer [µM]	MgCl_2 _[mM]	°Tm	Expected size	Range size observed			
SSR1	AGAGGACTCCATTTGTTTGCT	TGAGGGTGTTGGATGTTTTCT	0,2	2	50	206	170	210	AT	3′ UTR
SSR2	CATTGGGTTTCGCATCCAT	AGACAAGCCTAGGGGAAAGG	0,2	2	50	237	230	250	AG	3′ UTR
SSR9	TGCTCCGAGTTTTAGGGTTC	GCAGTTGGTAAAGTTGAGAGACG	0,2	2	50	193	220	240	AG	5′ UTR
SSR10	GCTTCCTATTGTGTTGCCTGA	ACTTTGGGTTTCGGGAATTG	0,2	2	50	185	170	190	AT	3′ UTR
SSR11	CAGCTGAAATAAGAGAGTGATTGG	CCCTCTTTTTCTCCTCCGAGT	0,2	2	50	180	180	210	AG	3′ UTR
SSR13	GCGGAATCCATTGTTTTTCA	CCGATGAGATATAGTCACGCAAA	0,2	2	50	190	160	210	AC	5′ UTR
SSR14	TGAAACCCATCTAGCTGAACG	TGGGTTGTTCCTTACAATCCAT	0,2	1,5	50	204	200	220	AT	3′ UTR
SSR15	GCTTGTTGATCAGCTTTCTTTG	TGGATCATAACCTTGCTAATGC	0,2	1,5	50	172	160	180	AT	3′ UTR
SSR18	CAGAGTGATTACCTTGGACGAA	TGTCCATTTTAGTCGCCAAT	0,2	1,5	50	179	180	230	AC	3′ UTR
SSR20	GCACATCACATAAAGTATCTTTCTCA	TTGCCTGGTGTCTTGCTATG	0,2	1,5	50	270	170	220	AT	3′ UTR
SSR36	ATGAACCACATGTCGGAGGA	GGGGATCCAAACGAAGTGTA	0,2	1,5	52	211	170	240	AG	3′ UTR
SSR37	CCAACTGAATCAACACACAGC	CCACACTGAAAAAGGGATCTG	0,3	2	50	212	260	330	AG	3′ UTR
SSR54	CGGCTGGTATGCTTACAAAGAT	GCACTTCCACTGTTTTTAACTTCC	0,2	1,5	50	197	190	210	AC	3′ UTR
SSR55	CACCTACATAGGCAGCCAAAA	ATTTGTGGGCGGAGGAAG	0,2	1,5	50	183	200	210	AG	5′ UTR
SSR57	AGTGAAAAGCAGCCCATTCT	GGCGAAGCTGAATTGAAAAA	0,2	1,5	50	183	200	210	AT	3′ UTR
SSR67	GCTTCTGTTCCATTATTCACCA	GCAGTGTGGGATCAATCAAT	0,2	1,5	50	207	180	240	AG	3′ UTR
SSR68	GAAGCAAACAACTACACCCAAA	AAGCCTCGGATTTCATAGCA	0,2	1,5	50	187	160	220	AG	3′ UTR
SSR72	GTGCTCGCAGTTTCTTCAAA	CCGCCGTTACTTCCTAATCA	0,2	1,5	50	158	130	170	AG	3′ UTR
SSR77	CATACCATAACTCCCCATCTCTC	TGCCGATTCTGATTTCTTCC	0,2	1,5	50	216	170	200	AT	5′ UTR
SSR92	TGGTTTGAGGATCAAGAAAGAA	GTGGTATCAACGCAGAGTGG	0,25	2,5	50	205	180	210	AAG	3′ UTR
SSR107	CATCCAACACCAGAAATACGC	TCCAACTTTATCATTTCTTCCAC	0,2	1,5	50	206	220	250	AAG	5′ UTR
SSR110	CACCCATATCCCAATCTTCTTC	GGGTAATTTTCACGGGGAAT	0,2	1,5	50	198	170	200	CTT	3′ UTR
SSR112	CTACGCCTACCACTTGCACA	CAGTGGAAGCCTCAAGATCC	0,2	1,5	50	203	200	220	TCT	3′ UTR
SSR118	AATCAAGGGTCAGAAGAAATGG	GCAAGAATGGATGTGGGTGT	0,2	1,5	50	180	130	180	AAG	5′ UTR
SSR121	AGCAACCTCCCAATCAGCTA	TGGTGAGTAAATGGGGGAAA	0,2	1,5	50	189	170	190	ATC	3′ UTR
SSR123	TCAGTGGAGCGCGTATATCT	GCGATCTCACCAAACCTCTC	0,2	1,5	50	216	190	210	ATC	5′ UTR
SSR126	TCCAAAAAGAAAACAAAAACACT	TTGAATGCATGTTTGATGGA	0,2	1,5	50	202	190	200	AGC	5′ UTR
SSR127	TTGGTTTGGCATAACTGCAA	GGTTTGCAACTCTCATGCTG	0,2	1,5	50	180	140	160	AAT	5′ UTR
SSR138	TCCGATCACTACTTCAGCACG	CAATTCGGGTTGTGAATCGGGT	0,2	1,5	50	138	130	160	AAT	3′ UTR
SSR146	AGGCTAATGAGGACGAAGCA	GGTTGCATTACAAAGCACTGA	0,2	1,5	50	187	160	210	AAAAG	3′ UTR

### Functional relationships of polymorphic SSR markers

A significant GO annotation was found for 10 of the 30 markers, which are related to 43 different ontology terms, of these 27 (67%) were related to biological process, 11 (25%) to molecular function and 5 (8%) to cellular component (Table 5).

**Table 5:** Functional annotation of 10 *P. peruviana* contigs containing polymorphic SSR markers.

SSR Marker	GO Category: ID	Functional Annotation
SSR2	P:0006350	Transcription
SSR37	F:0016301	Kinase activity
	C:0005886	Plasma membrane
SSR54	P:0006952	Defense response
	P:0012501	Programmed cell death
	C:0044464	Cell part
	F:0000166	Nucleotide binding
SSR55	P:0051865	Protein autoubiquitination
	F:0004842	Ubiquitin-protein ligase activity
	P:0048437	Floral organ development
	P:0046621	Negative regulation of organ growth
SSR77	P:0009789	Positive regulation of abscisic acid mediated signaling pathway
	P:0006979	Response to oxidative stress
	P:0052544	Callose deposition in cell wall during defense response
	P:0009753	Response to jasmonic acid stimulus
	P:0031348	Negative regulation of defense response
	P:0008219	Cell death
	P:0009651	Response to salt stress
	P:0042742	Defense response to bacterium
	P:0009926	Auxin polar transport
	P:0010119	Regulation of stomatal movement
	P:0009408	Response to heat
	F:0005515	Protein binding
	P:0010150	Leaf senescence
	P:0048765	Root hair cell differentiation
	P:0009871	Jasmonic acid and ethylene-dependent systemic resistance, ethylene mediated signaling pathway
	P:0001736	Establishment of planar polarity
	P:0050832	Defense response to fungus
	P:0010182	Sugar mediated signaling pathway
SSR92	F:0004674	Protein serine/threonine kinase activity
	P:0045449	Regulation of transcription
	P:0007169	Transmembrane receptor protein tyrosine kinase signaling pathway
	F:0005524	ATP binding
	F:0003700	Transcription factor activity
	P:0010030	Positive regulation of seed germination
	P:0006468	Protein amino acid phosphorylation
SSR110	C:0044444	Cytoplasmic part
SSR126	F:0005488	Binding
	F:0003824	Catalytic activity
SSR138	F:0016740	Transferase activity
SSR146	C:0005730	Nucleolus
	C:0016020	Membrane
	F:0003677	DNA binding

Gene ontology (GO) functional Categories: **C** = Cellular component, **F** = Molecular function, **P** = Biological process.

## Discussion

Here we present the first collection of EST-derived microsatellite markers in *Physalis peruviana*. The highest number of SSR loci found contained trinucleotide and hexanucleotide repeats (Table 2), which is consistent with results reported in Solanaceae and other plant species [19], [20], [25], [26], [27], [28], [29], [30], [31]. 1,236 out of 1,520 SSR loci are composed of imperfect repeats increasing the probability of polymorphism among *Physalis* species. This inference is bolstered by the fact that 30 of the 162 imperfect SSRs (22%) were polymorphic in the panel of 8 accessions from *P. peruviana* and the related species *P. floridana* (Table 1), suggesting the potential utility of these genetic based SSR markers for future studies. i.e. germplasm diversity and breeding applications [17], [19], [32].

Our results show that most of the SSR loci were located at UTRs (Table 2) in agreement with the results reported by Morgante and others [27] who hypothesize that in plants most of the SSR loci from transcribed regions are distributed along the UTRs. Increased numbers of SSR loci at UTRs could be related to changes in transcription (5′UTRs) or RNA silencing (3′UTRs), which are sources of variation among species [18], [19], [20], [29], [30]. Cereal species appear to have a different SSR distribution; Yu and others [33] found that most of the 444 EST derived SSR markers (62%) were located at coding regions, while 38% were located at UTRs.

Since the SSR loci found in this study were derived from genes, they may be related to some traits of interest [18], [20], [27] such as resistance to *Fusaruim oxysporum*, which is one of the main constraints for Cape gooseberry production at the commercial level. According to the functional annotation obtained by the GO analysis, two polymorphic SSR markers (SSR54 and SSR77 respectively) were related with proteins involved in defense responses to pathogens such as programed cell death and ethylene as well as jasmonic acid pathways. These two polymorphic SSR makers would be useful in *P. peruviana* breeding programs focused on *F. oxysporum* resistance.

The high rate of successful PCR amplification for the primer pairs designed (84%, Table 4) is related to the fact that these loci are specific to *P. peruviana* and they were also developed from genes, increasing the transferability within species of the same genus i.e. *P. floridana*. These results are in agreement with Zeng *et al*. and Csencsics *et al*. [19], [21], who used full-length cDNA and ESTs and found rates of successful PCR amplification larger than 80%.

This study reports the first set of microsatellite markers developed for *P. peruviana* and related species. A total of 1,520 SSR loci were identified, including 932 imperfect SSRs located at UTRs. From these loci a total of 162 SSR primers were developed to assay their utility as microsatellite markers in a panel of seven accessions of *P. peruviana* and one accession of *P. floridana* by PCR amplification. A total of 138 (83%) primer markers amplified, with a polymorphism rate of 22%. The markers developed here can be used in plant breeding programs that may ultimately lead to superior phenotypic characteristics such as increase in fruit size, reduction in the tendency to split during transport, reduction in the plant susceptibility to pests and diseases, and improvement of fruit quality.
